# The Effect of Core Stabilization Training on Improving Gait and Self-Perceived Function in Patients with Knee Osteoarthritis: A Single-Arm Clinical Trial

**DOI:** 10.3390/pathophysiology29030040

**Published:** 2022-09-01

**Authors:** Daniel W. Flowers, Wayne Brewer, Katy Mitchell, Jennifer Ellison, Clifton Frilot

**Affiliations:** 1 Program in Physical Therapy, LSU Health Shreveport, 1501 Kings Hwy, Shreveport, LA 71103, USA; 2 School of Physical Therapy, Texas Woman’s University, 6700 Fannin, Houston, TX 77030, USA

**Keywords:** kinetics, kinematics, core musculature, physical therapy, walking

## Abstract

The treatment of patients with knee osteoarthritis is typically focused on the involved lower extremity. There is a gap in the literature concerning the effectiveness of core stabilization training on the treatment of patients with knee osteoarthritis. This investigation aimed to determine whether core stabilization improved the gait and functional ability of patients with knee osteoarthritis. Eighteen participants with knee osteoarthritis completed the six-week core stabilization intervention. Participants completed the gait motion analysis and the Knee Injury and Osteoarthritis Outcome Score to assess self-perceived function, pre- and post-intervention. Gait speed improved (*p* = 0.006, *d* = 0.59), while the external knee adduction moment decreased (*p* = 0.034, *d* = −0.90). Moreover, self-reported function improved (*p* < 0.001, *d* = 1.26). The gait speed and external knee adduction moment changes met minimal detectable change thresholds, while gait speed also met the minimal clinically important difference. A six-week core stabilization program can thus improve gait speed and reduce the external knee adduction moment, which is tied to disease progression. Increased functional scores post-intervention indicate an important clinical improvement. Core stabilization training is a safe and potentially effective treatment option for this population.

## 1. Introduction

Knee osteoarthritis (KOA) doubled in prevalence from 1990–2019, and accounts for approximately 60.6% of the total burden of osteoarthritis globally [1]. Risk factors for developing KOA include increased age and body mass index (BMI), in addition to being female [2]. Current clinical practice guidelines strongly recommend treatment with oral medications, land-based strengthening and cardiovascular training programs, and patient education [3]. Exercise prescription has been recognized for its ability to reduce KOA-related pain [4]. Recommendations for exercise interventions include aerobic training, strengthening exercises, aquatic therapy, and patient education on self-management and self-modification of their physical activity [5]. Physical and occupational therapy continue to be one of the most affordable treatment options for patients with KOA, accounting for only 2.0% of pre-total knee arthroplasty treatment costs [6]. Exercise and exercise-related education continue to rank among the highest recommendations established by experts for the treatment of KOA [7].

Limitations in gait commonly encountered by those with KOA include decreased gait speed [8,9] and alterations to the biomechanical aspects of gait. Abnormal mechanics include alterations in kinetics and kinematics, such as increased time to first peak ground reaction force (T1) [9]; decreased second peak ground reaction force (F2) [9] at terminal stance/pre-swing; alterations to the peak knee flexion angle in stance (KFA) [10]; and an increased knee adduction moment (KAM) [11,12,13].

A review of the literature found that the current evidence for the management of KOA is still primarily extremity-focused [14]. This review concluded that investigating core muscle contributions was warranted in the management of KOA given the lack of previous study and preliminary evidence of a link between KOA and the core muscles. Previous work indicates that those with KOA have decreased postural stability [15], increased trunk and pelvic movement [16], reduced core muscle endurance [17], and reduced abdominal muscle thickness [18]. A previous investigation found that core muscle activation had no effect on the gait of those with KOA; however, a core stabilization program was considered appropriate for future study given the possible effects that could result from formal training [9].

### Purpose and Hypotheses

The primary purpose of this preliminary investigation was to determine whether a six-week core stabilization program altered the function and gait kinetics, kinematics, and speed of patients with KOA. The secondary purpose was to determine whether there was a predictive relationship between the number of completed intervention sessions performed and the observed changes. The alternative hypotheses for this investigation were that, in participants with KOA, after a six-week core stabilization program: (1) gait speed, kinetics (T1, F2, and KAM), and kinematics (KFA) would significantly differ compared to baseline during self-selected paced ambulation; (2) functional ability, as assessed via the Knee Injury and Osteoarthritis Outcome Score (KOOS), would significantly differ; and (3) the total number of supervised and home exercise sessions would significantly predict any changes in gait observed.

## 2. Materials and Methods

### 2.1. Participants

The protocol for this single-arm clinical trial prospectively registered with ClinicalTrials.gov (NCT03776981). An a priori power analysis was performed using effect sizes from previous studies in the literature, with gait speed being the variable used for determination (*f* = 0.50) [19], and a sample size of 18 was required. A 20.0% attrition rate was assumed, which resulted in 22 participants being the goal for recruitment. Participants were recruited from the community and within the university system via hard-copy and digital flyer postings, word-of-mouth, and clinical referrals. The inclusion and exclusion criteria were the same as in our previous investigation [9]. The inclusion criteria required that participants be English-speaking men and women over the age of 40 of any race or ethnic group, with a documented diagnosis from a medical provider (i.e., physician, physician assistant, or nurse practitioner, etc.) of KOA, unilaterally or bilaterally. Previous and current physical therapy patients were included. Exclusion criteria included those with other lower extremity injuries (orthopedic, vascular, and decreased function secondary to neurological insult, etc.) that hindered their ability to ambulate; current complaints of lower back pain; those who had undergone bilateral total knee arthroplasty; concomitant diagnosis of rheumatoid arthritis; those who were unable to ambulate independently with or without an assistive device; those who had received a corticosteroid injection within the past two months; those who had received a hyaluronic acid injection in the past six months; those who were currently enrolled in a core training program as part of formal physical therapy or physical fitness. No current therapy patients were recruited for this study.

### 2.2. Instrumentation

The kinetic (T1, F2, and KAM), kinematic (KFA), and gait speed data were collected via a Vicon Vero 2.2 Motion Analysis System (Vicon, Oxford, UK) sampling at 100 Hz integrated with two force plates (Advanced Medical Technology, Inc., Watertown, MA, USA) with a sampling rate of 2 kHz. 

The KOOS is an outcome measure commonly employed in clinical environments when evaluating patients with KOA [20]. The overall score, the sum of each of the five subsections out of 500, is correlated with the Kellgren–Lawrence scale, which grades KOA severity [21]. The KOOS questionnaire was used in order to collect information on the self-perceived functional ability of the participants. The questionnaire was completed by the participants prior to gait analysis before and after the intervention, with the overall score totaled by the principal investigator (PI).

### 2.3. Procedures

After obtaining informed consent, all participants first underwent anthropometric data collection, including weight, height, leg length, and knee and ankle joint width. Participants completed the KOOS questionnaire and provided informed consent simultaneously. Reflective markers were placed on bilateral lower extremities (bilateral anterior and posterior iliac spines, lateral thighs, knee lateral epicondyles, lateral legs, lateral malleoli, calcanei, and second metatarsals). All marker placements on participants were performed by the PI. The Vicon system underwent calibration, and a static trial was completed. For participants with bilateral KOA, the most painful limb, identified by subjective report, was used for data collection. 

The participants were allowed a brief walking warm-up of one to two minutes prior to data collection if desired. Participants were instructed to walk at a self-selected pace across the capture area until three trials with satisfactory force plate contact were obtained. Satisfactory force plate contact was defined as initial contact at the heel and pre-swing/toe off occurring on the force plate, and the absence of any evidence of distraction via the video cameras, such as the participant talking or looking around the lab. 

Participants received a six-week core stabilization intervention program (Table S1: Intervention Program). The program was developed using exercises from previous authors [22,23]. One previous, underpowered investigation included a 12-week intervention to compare typical treatment to typical treatment with a core training component for patients with KOA [24]. Our investigation employed a six-week intervention based upon previous recommendations for non-pharmacologic interventions for KOA; recommendations included a dosage of two exercise sessions for six weeks, with one to two additional sessions per week for optimal effect [5]. This program commenced within seven days of the initial gait data being collected. Participants attended two scheduled, supervised intervention sessions each week, of 20–30 minute duration. This duration of single sessions falls within the range commonly observed in other studies [25]. Additionally, the intervention program consisted of exercises from the supervised program performed at home independently. Participants were provided a handout (Table S2: Home Exercise Program) and education on these exercises by the PI. These were performed three times per week, every other day, and not on the same day as a supervised intervention session. The handout included boxes participants checked each time they completed a home exercise session to track compliance. The supervised intervention sessions were performed in an outpatient physical therapy clinic. With the participants’ consent, up to three participants underwent the intervention with the PI simultaneously. Supervised training sessions were never performed on consecutive days in an effort to reduce the development of delayed onset muscle soreness. Additionally, the core training program included predetermined guidelines on exercise progression to ensure the exercises did not progress too rapidly. The PI led all sessions to ensure the exercises and their progressions were conducted correctly. Progression of the exercises within each week of the program was based upon individual ability, residual soreness, and ability to perform each exercise correctly and safely. Participants were removed from the study if they missed more than four supervised sessions, or did not perform the home program at least once a week. Their data were not analyzed.

After the completion of the six-week program, participants completed a second, post-intervention KOOS questionnaire, and turned in their home exercise program sheet with tallies from completed sessions. Participants then underwent a post-intervention gait analysis using the same methodology as in the pre-test.

### 2.4. Data Processing

The gait data were processed using the Plug in Gait model within the Nexus (Vicon, Oxford, UK) processing system. Missing marker trajectories were filled with maximum gap length, empty, and cyclic filling prior to processing the model. These data from the three trials were averaged, with means used for data analysis. The KOOS and the total number of supervised and home sessions completed were calculated and tallied by the PI, respectively.

### 2.5. Data Analysis

There were missing data points from two participants’ pre-intervention KFA and KAM data, and another participant’s post-intervention KFA and KAM data. This accounted for only 3.4% of the total biomechanical data (T1, F2, KFA, KAM, and gait speed), with only six out of 180 data points missing. These data were considered missing completely at random (MCAR) since it was related to hardware/software issues. Subgroup mean substitution was used for these missing values [26,27] because it is preferred to overall mean substitution, which reduces the variance of the data [27,28]. Mean substitution is also better able to approximate the value of the missing data points [28], and is less likely to cause bias when the missing data is MCAR as compared to that missing at random or not at random [29].

## 3. Results

**Figure 1:**
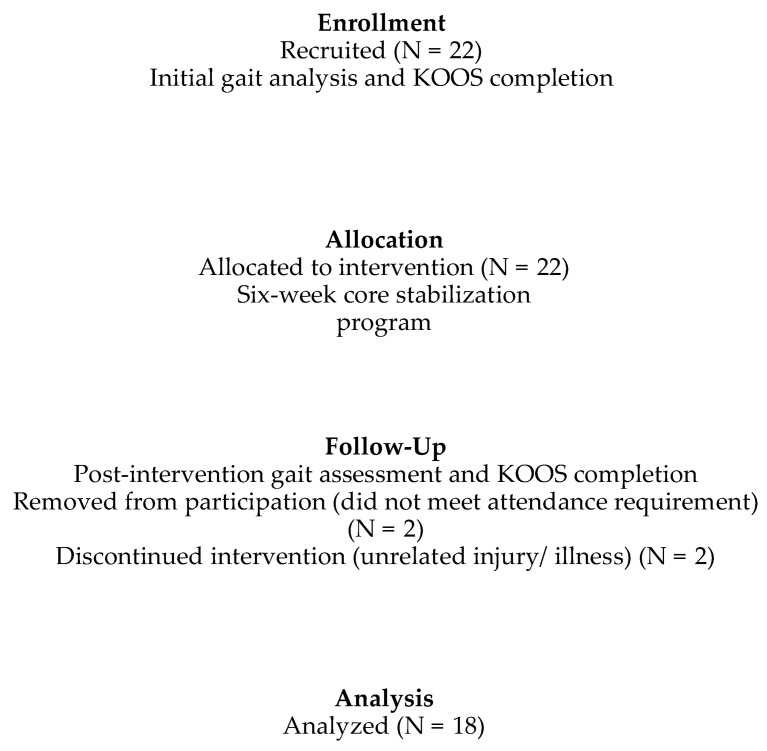
Study flowchart.

From January 2019 until July 2019, 22 participants were recruited, five males and 17 females. Four participants did not complete the six-week intervention program (see Figure 1), leading to a final sample of 18 (five male and 13 female) participants (see Table 1).

### 3.1. Gait Analysis

**Figure 2:**
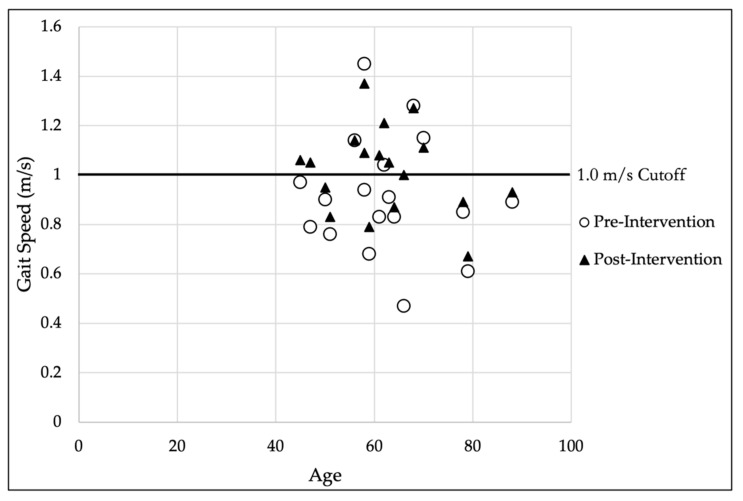
Pre- and post-intervention gait speed versus participant age.

To test the first hypothesis, paired-sample t-tests were performed for each dependent variable to examine the within-subject effects pre- and post-intervention for the gait analysis variables (see Table 2). Medium to large effect sizes (Cohen’s d) were observed for all significant differences observed. Figure 2 presents the gait speed pre- and post-intervention versus participants’ age. Table 3 provides the changes in gait speed pre- and post-intervention by decade of age.

### 3.2. KOOS Analysis

In testing the second hypothesis, a paired-sample t-test was performed to analyze the pre- and post-KOOS to determine whether a significant change had occurred post-intervention. A significant improvement was observed in the overall KOOS when compared to the pre-intervention (M = 253.0 (70.81)) and post-intervention (M = 342.0 (66.75)) percentage scores, 95% CI (−114.49 to −63.64), *t*(17) = −7.39, *p* < 0.001, with a large-sized effect, *d* = 1.26.

### 3.3. Dose–Response Analysis

To test the third hypothesis, both the number of supervised visits and home exercise program sessions completed were totaled. This total number of intervention sessions was entered as the predictor variable in two separate linear regression analyses. These two analyses used the amount of change in KAM and gait speed between the pre- and post-intervention as the outcome variables of one of each regression analysis, since they were observed to exhibit significant changes post-intervention. There was no significant predictive relationship observed between the total number of exercise sessions (M = 21.14 (10.20)) and the change in KAM (M = 0.18 (0.34)), *r* = 0.076, *p* = 0.77, or in gait speed (M = 0.079 (0.094)), *r* = 0.035, *p* = 0.90.

### 3.4. Correlation Analysis

**Figure 3:**
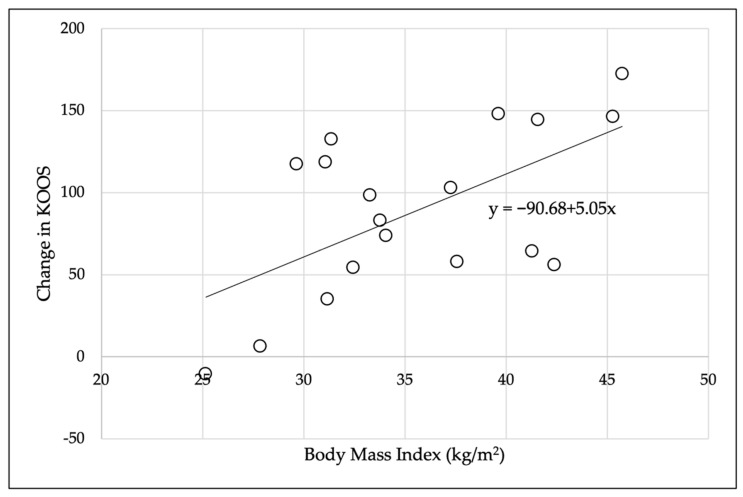
Relationship between body mass index and change in KOOS.

To evaluate the effect of BMI, correlation analyses were run between BMI and the amount of change in each dependent variable pre- and post-intervention for each participant who completed the intervention program. Table 4 provides the results of the analyses, and Figure 3 provides details of the significant positive relationship between BMI and change in the KOOS. 

## 4. Discussion

For the first hypothesis, the gait analysis revealed there was a significant change in both gait speed and the KAM after a six-week core stabilization program. While the gait speed increased, the KAM decreased. Not only was there a statistical difference, but a clinical difference as well. Specifically, the average gait speed improvement of 0.10 m/s meets both the minimal detectable change (MDC) [30] and minimal clinically important difference (MCID) [31] thresholds of at least 0.10 m/s. Apart from the MDC and MCID, an improvement in gait speed of 0.10 m/s also predicts improved life expectancy [32,33]. Furthermore, the group’s pre-intervention average gait speed of 0.92 m/s fell below the established cutoff of 1.0 m/s, below which, functional decline arises [34,35]. Considering the average age for this sample of participants was 62.39 years, an age group that has normative gait speed of 1.34 m/s for men and 1.24 m/s for women, this finding takes on even more clinical importance. Caution should be taken when applying these normative data to this specific sample, however, given the age range for the norm data is 60–69 years [36], whereas this study presented an average from a range of 43 years (45–88 years of age). Additionally, the amount of change in gait speed varied across age-groups, with greater change observed in those in their fifth and seventh decades as compared to the others (Table 3). Regardless, at a gait speed of 0.92 m/s, cognitive decline, increased fall risk, increased rates of lower extremity limitations, and even death have been observed [35]. Those with a gait speed of at least 1.0 m/s have also been shown to have improved life expectancy when compared to norms expected for someone of the same age and sex [33]. In this study, the average age of those falling below and above the 1.0 m/s cutoff pre-intervention was 62.23 and 63.80 years, respectively. The average age of those who were below the cutoff pre-intervention and were above post-intervention was 56.67 years. This suggests that the improved average gait speed of the group may, if it can be reproduced in follow-up randomized controlled trials, provide use in determining whether this new intervention can help improve functional ability and overall health in this population. 

The KAM decreased by 0.18 Nm/kg after the six-week training program. Similar to gait speed, this change also met the established MDC of 0.17 Nm/kg (95% CI), converted from the value of 1.0% bodyweight x height previously established [37]. These authors noted this value to be helpful in determining whether a given intervention led to decreased compressive load of the medial joint space. The suggested mechanism for this reduction in KAM is that improved postural stability at the trunk improves the function of the arthritic knee. This proposal aligns well with previous findings [16,38,39]. Although measuring these biomechanical mechanisms at the pelvis and hip is beyond the scope of this investigation, this component is critical to future research in this area. Similar to gait speed improvement, this reduction in KAM has clinical implications. Increased KAM is observed with KOA progression, with authors advocating for clinical trials aimed at modifying this risk factor [40]. One systematic review found that gait modifications (e.g., increased and decreased toe-out, increased and decreased gait speed, etc.) have been explored as mechanisms to reduce the KAM [41]. Most of the studies’ interventions were aimed at altering natural gait patterns. One investigation examined the predictive relationship between gait speed and the KAM, finding there was no consistent pattern due to a wide variation within the sample; however, it did advocate for reducing the KAM by reducing gait speed, especially in the earlier stages of disease progression [42]. This point is further supported by a review of the in vivo progression of KOA [43]. Considering this recommendation, in addition to the various ones previously explored [41], one can see the conflict between the evidence put forth previously on the importance improving gait speed and the concurrent need to reduce the KAM. Although the design of this present study prevents a conclusion on the causative effect of core stabilization training on improved gait speed and decreased KAM, follow-up studies could indicate whether these two benefits could be achieved simultaneously. Such findings would not require the patient volitionally to alter their gait, resulting in an immediate progression to a mental dual-task activity by allowing self-selected speed after core stabilization training. Although gait mechanics are changed, they would be dependent upon compensatory mechanisms.

Overall, the observed changes to the KAM and gait speed in the individuals post-intervention were beneficial. This was not the case in the authors’ previous investigation, where core muscle activation while walking was not sufficient to elicit gait changes [9]. The authors cannot rule out possible improvements in other factors related to gait that this program may have impacted (but not measured) such as balance, hip strength, muscle recruitment, etc. Balance issues can lead to increased risks of falls or functional decline, both of which can be predicted by gait speed [35]. The increased medial knee joint compression resulting from a contralateral hip drop can be overcome by improving the strength of the ipsilateral hip abductor [44]. Additionally, activation patterns of the core muscles change with varied walking speeds [45], indicating the way the core muscles are recruited or used may alter gait. 

This investigation found a significant improvement in the overall KOOS after the participants completed the six-week intervention. Although no MCID has been established for the KOOS, the large effect size observed in this investigation is typical of other patient responses to treatment, including: anterior cruciate ligament reconstruction, microfracture, partial meniscectomy, and the response of patients with KOA to physical therapy [46]. The KOOS provides valuable clinical information for therapists in developing a plan of care. Of the 18 participants who completed the intervention program, all but one saw an improvement in their KOOS. One interesting finding was the positive correlation between BMI and change KOOS, indicating that those with a higher BMI saw greater improvements in their functional ability. There were no long-term follow-ups in this investigation, so it is unknown whether these gains were maintained over time. A randomized controlled trial examining females with KOA found that yoga significantly improved scores on the pain, activities of daily living, sport and recreation, and quality of life subscales of the KOOS [47].

Previous work showed a significant correlation between KOA severity and overall KOOS [21]. A large increase in KOOS as observed in this investigation, when paired with the reduced KAM, may indicate altered loading and patient-reported outcomes and should be explored in future studies. Since there were no adverse events associated with the intervention program, a core stability program is safe and feasible in this patient population, when considering both average age and pathology. As no long-term follow-up was used in this study, the feasibility of this intervention as a definitive intervention to maintain functional gains cannot be established. This preliminary investigation observed changes in biomechanical factors associated with disease propagation (i.e., KAM), improved gait speed which is associated with overall health status, and improvements in patients’ perceived functional ability after participants underwent a core stabilization program. Therefore, this type of intervention warrants further study. Specifically, core stabilization training should be prescribed in appropriately powered randomized controlled trials prior to being included in the standard of care for treating patients with KOA.

To our knowledge, there is only one other clinical trial, that by Hernandez et al. [24], that has investigated the effect of core muscle training on functional recovery in patients with KOA. This randomized controlled trial compared a traditional exercise intervention program to one which included traditional exercises and core muscle training. Pain, assessed via the visual analog scale, was s significantly lower in short-term follow-ups in the group treated with core exercise than in those without core muscle training. However, there was no difference in pain at long-term follow-ups. The investigators also examined function, using the Timed Up and Go Test, the Step Test, and the functional component of the Western Ontario and McMaster Universities Arthritis Index (WOMAC). No differences were observed between the groups, but significant improvement was observed in both groups over time. The investigation contained nine participants short of the N needed to achieve adequate power per their own a prior power analysis. The authors did not investigate the biomechanical variables that were the focus of our investigation.

Future studies should explore optimal dosing, exercise prescription [48], and the need for supervision as they relate to this new intervention [25], in addition to exploring the causative nature of the therapeutic effect. Long-term follow-up should also be included to determine the ability of core stabilization to allow individuals to maintain their functional gains over time.

### Limitations

There are several limitations noted in this investigation. First, the severity of KOA was not directly assessed or controlled for in this investigation. Therefore, these results cannot be applied to any particular severity grade(s) of KOA. Second, as with all motion analysis studies, there is the issue of possible marker placement error. This was minimized by having only the PI place the markers. Third, also related to marker placement, is the high BMI observed in this sample. Since increased adipose tissue can cause alterations in exact marker positions, this should be considered when analyzing the results of the biomechanical data. Fourth, the vast majority (77.3%) of participants were female, therefore this too should be considered when applying these results clinically. Fifth, the lack of long-term follow-ups prevents the investigators knowing whether the improvements observed were maintained over time. Finally, there was no control group in this investigation, so comparing the outcomes of this core stabilization intervention to a standard of care treatment or no treatment is not possible. 

## 5. Conclusions

In conclusion, this investigation demonstrated that a six-week core stabilization program increases walking speed, reduces the KAM, and improves functional ability as measured via the KOOS for individuals with KOA in a single-cohort repeated measures design. 

## Figures and Tables

**Table 1:** Descriptive data for participants.

Variable	Mean (SD)	Minimum	Maximum
Age (years)	62.39 (11.36)	45	88
Height (m)	1.65 (0.08)	1.56	1.84
Weight (kg)	97.20 (19.39)	61.36	127.73
BMI (kg/m^2^)	35.58 (6.03)	25.15	45.75
Leg Length (cm) ^a^	87.14 (5.22)	78	101
Knee Width (cm) ^a^	11.77 (0.94)	10.30	13.50
Ankle Width (cm) ^a^	7.18 (0.55)	6.40	8.40

Abbreviations: SD, standard deviation; BMI, body mass index. ^a^ Values provided are for the affected/assessed lower extremities.

**Table 2:** Results of pre- and post-treatment repeated measures t-tests for biomechanical data.

Variable	Pre-Intervention ^a^	Post-Intervention ^a^	Group Differences
T_1_ (%ST)	30.45 (6.31)	27.78 (5.72)	*p* = 0.051, *d* = −0.47, 95% CI (−0.01 to 5.36)
F_2_ (%BW)	99.27 (5.11)	100.28 (4.03)	*p* = 0.27, *d* = 0.25, 95% CI (−2.85 to 0.84)
KFA (°)	30.41 (9.21)	33.54 (5.50)	*p* = 0.12, *d* = 0.34, 95% CI (−7.12 to 0.87)
KAM (Nm/kg)	0.68 (0.35)	0.50 (0.20)	*p* = 0.034 ^b^, *d* = −0.90, 95% CI (0.02 to 0.35)
Gait Speed (m/s)	0.92 (0.24)	1.02 (0.17)	*p* = 0.006 ^b,c^, *d* = 0.59, 95% CI (−0.17 to −0.03)

Abbreviations: T1, time to first peak ground reaction force; %ST, percent stance time; F2, second peak ground reaction force; %BW, percent body weight; KFA, max knee flexion angle in stance; KAM, knee adduction moment. ^a^ Values given are group means, with standard deviations in parentheses. ^b^ Significant at alpha = 0.05. ^c^ Significant at alpha = 0.01, with Bonferroni applied.

**Table 3:** Change in gait speed across pre- and post-intervention time points by age.

Age (Years)	*N*	Minimum (m/s)	Maximum (m/s)	Mean (m/s)
40–49	2	0.1	0.25	0.18
50–59	6	−0.08	0.15	0.05
60–69	6	−0.02	0.53	0.19
70–79	3	−0.04	0.06	0.02
80–89	1	NA	NA	0.04

Abbreviations: NA, not applicable.

**Table 4:** Correlations between body mass index and amount of change in dependent variables.

Variable	Correlation/ Significance	BMI	T1 Change	F2 Change	KFA Change	KAM Change	Gait Speed Change	KOOS Change
BMI	*r*	1						
	*p*							
T1 Change	*r*	0.40	1					
	*p*	0.10						
F2 Change	*r*	−0.041	−0.40	1				
	*p*	0.87	0.10					
KFA Change	*r*	−0.22	0.18	0.12	1			
	*p*	0.37	0.47	0.65				
KAM Change	*r*	0.18	0.30	−0.042	0.31	1		
	*p*	0.46	0.23	0.87	0.21			
Gait Speed Change	*r*	0.055	0.31	−0.45	−0.09	−0.091	1	
	*p*	0.83	0.21	0.059	0.72	0.72		
KOOS Change	*r*	0.60 ^a^	0.17	−0.14	−0.001	0.085	0.38	1
	*p*	0.009 ^a^	0.50	0.58	0.10	0.74	0.12	

Abbreviations: BMI, body mass index; T1, time to first peak ground reaction force; F2, second peak ground reaction force; KFA, max knee flexion angle in stance; KAM, knee adduction moment; KOOS, Knee Injury and Osteoarthritis Outcome Score. ^a^ Significant at alpha = 0.01.

## Data Availability

The data presented in this study are available on request from the corresponding author.

## Supplementary Materials

The following supporting information can be downloaded at: https://www.mdpi.com/article/10.3390/pathophysiology29030040/s1. Table S1: Intervention Program; Table S2: Home Exercise Program.
