# Supplementary material for: The Effect of Core Stabilization Training on Improving Gait and Self-Perceived Function in Patients with Knee Osteoarthritis: A Single-Arm Clinical Trial
Source: Pathophysiology. 2022 Sep 1;29(3):495–506. doi: 10.3390/pathophysiology29030040 (PMC9505132; doi:10.3390/pathophysiology29030040)
Supplement: Supplementary file 1 [file pathophysiology-29-00040-s001.zip › Supplementary Table S1_Intervention Program.pdf]

CORE STABILIZATION PROGRAM

Week	Exercises	Progression/Intensity
1	1. Transverse abdominis contraction education/performance‖<br>2. Supine bent knee fall outs*‖<br>3. Dead bug alternating leg lifts*‖<br>4. Quadruped alternating arm lifts*<br>5. Quadruped alternating leg slides*<br>6. Curl ups<br>7. Bridge with alternating leg extension	1. Initiate in side-lying, progress to supine.<br>2. NA<br>3. Flexed knee moving leg → Sliding moving leg → straight leg raises on moving leg<br>4. Add exercise 4 and 5 together as alternating arm and leg movements.<br>5. See 4.<br>6. NA<br>7. Add transverse abdominis contraction to the exercise.
2	1. Transverse abdominis contractions‖<br>2. Dead bugs with one hip held by hand in 90 degrees of flexion, alternating leg lifts*‖<br>3. Quadruped hip extensions*‖<br>4. Quadruped alternating shoulder flexion/hip extension*<br>5. Curl ups<br>6. Bridge with alternating leg extension*	1. 3 sets x 10 reps<br>2. Flexed knee moving leg → Sliding moving leg → straight leg raises on moving leg†<br>3. Add ankle weights as able.<br>4. Add ankle weights as able.
3	1. Transverse abdominis contractions seated on BOSU ball‖<br>2. Seated on BOSU, alternating marching*‖<br>3. Dead bugs with one hip held by hand in 90 degrees of flexion, alternating leg lifts*‖<br>4. Dead bugs with one hip actively maintained in 90 degrees of flexion, alternating leg lifts* (as able, as a progression from exercise 3)<br>5. Quadruped hip extensions with ankle weights*<br>6. Quadruped alternating shoulder flexion/hip extension with ankle weights*<br>7. Curl ups<br>8. Bridge with alternating leg extension, with ankle weights*	1. 3 sets x 10 reps<br>2. 3 sets x 10 reps<br>3. Flexed knee moving leg → Sliding moving leg → straight leg raises on moving leg†<br>4. Flexed knee moving leg → Sliding moving leg → straight leg raises on moving leg†<br>5. 3 sets x 10 reps‡<br>6. 3 sets x 10 reps‡<br>7. 3 sets x 10 reps<br>8. 3 sets x 10 reps‡

4	1. Seated on BOSU, alternating marching*‖<br>2. Seated on BOSU, alternating knee extensions*‖<br>3. Dead bugs with one hip actively maintained in 90 degrees of flexion, alternating leg lifts*‖<br>4. Prone alternating hip extensions<br>5. Seated on mat, bilateral symmetrical D2 flexion extension<br>6. Modified prone, and bilateral lateral planks<br>7. Curl ups	1. 3 sets x 10 reps<br>2. 3 sets x 10 reps<br>3. Flexed knee moving leg → Sliding moving leg → straight leg raises on moving leg†<br>4. 3 sets x 10 reps‡<br>5. 3 sets x 10 reps, with resistance as able<br>6. 2 sets to fatigue for all exercises, progress to 3 sets as tolerated at second visit<br>7. 3 sets x 10 reps
5	1. Seated on BOSU, alternating knee extensions*<br>2. Seated on BOSU, alternating shoulder flexion and march on opposite arm/leg*‖<br>3. Seated on BOSU, bilateral symmetrical D2 flexion extension*‖<br>4. Dead bug with both hips moving simultaneously through movements*‖<br>5. Prone alternating hip extensions with ankle weights<br>6. Prone bilateral, symmetrical hip extensions<br>7. Prone baby-Supermans<br>8. Modified prone, and bilateral lateral planks	1. 3 sets x 10 reps, with addition of ankle weights as tolerated<br>2. 3 sets x 10 reps<br>3. With resistance band as able.<br>4. Flexed knee lifts → Sliding leg extensions → straight leg raises†<br>5. 3 sets x 10 reps, increase weight as tolerated<br>6. 3 sets x 10 reps, add weight as able<br>7. 3 sets x 10 reps<br>8. Progress away from modification as tolerated, or increase to three sets. At least 2 sets to fatigue in each direction.
6	1. Seated on BOSU, alternating shoulder flexion and march on opposite arm/leg*<br>2. Seated on BOSU, bilateral asymmetrical D1/D2 flexion/extension*‖<br>3. Dead bug with both hips moving simultaneously through movements*‖<br>4. Prone bilateral, symmetrical hip extensions with ankle weights<br>5. Prone baby-Supermans with hand weights (as tolerated)<br>6. Prone Supermans‖<br>7. Modified or non-modified (as able) prone, and bilateral lateral planks	1. 3 sets x 10 reps, with addition of ankle weights as tolerated<br>2. 3 sets x 10 reps<br>3. Flexed knee lifts → Sliding leg extensions → straight leg raises†<br>4. 3 sets x 10 reps, increase resistance as able<br>5. 3 sets x 10 reps, add resistance as able<br>6. 3 sets x 10 reps<br>7. 3 sets to fatigue in each direction.

*These exercises will be performed while the patient maintains a transversus abdominis contraction.
†One set of each progression as able.
‡Increase weight on exercises as tolerated.
||Exercises to be performed independently, every other day three times weekly as a home exercise component for each week of the program. Exercises requiring equipment will be performed without in the home setting. Details provided in the Home Exercise Program Handout

References:

Kisner C, Thorp JN. The Spine: Exercise and Manipulation Interventions. In: Kisner C, Colby LA, eds. *Therapeutic Exercise: Foundations and Techniques.* 6th ed. Philadelphia, PA: F.A. Davis Company; 2012.

Granacher U, Lacroix A, Muehlbauer T, Roettger K, Gollhofer A. Effects of Core Instability Strength Training on Trunk Muscle Strength, Spinal Mobility, Dynamic Balance and Functional Mobility in Older Adults. *Gerontology*. 2013;59:105-113.
