# Supplementary material for: The Effect of Core Stabilization Training on Improving Gait and Self-Perceived Function in Patients with Knee Osteoarthritis: A Single-Arm Clinical Trial
Source: Pathophysiology. 2022 Sep 1;29(3):495–506. doi: 10.3390/pathophysiology29030040 (PMC9505132; doi:10.3390/pathophysiology29030040)
Supplement: Supplementary file 1 [file pathophysiology-29-00040-s001.zip › Supplementary Table S2_HomeExerciseProgram.pdf]

HOME EXERCISE PROGRAM

PARTICIPANT HANDOUT

please place a mark in each box associated with a day upon completing a session. if a session is not completed, leave a box empty.

**week**	**exercises**
1<br><br>• day 1<br>• day 2<br>• day 3	1. transverse abdominis contraction<br>2. supine bent knee fall outs<br>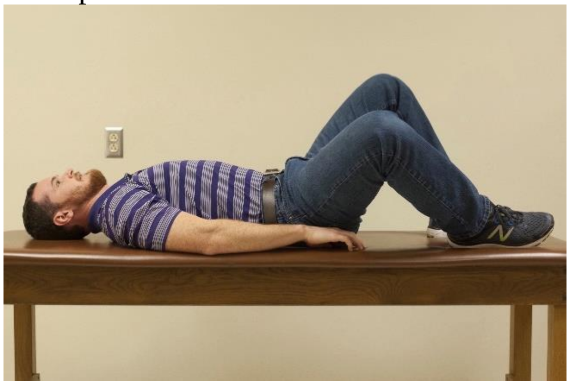<br>3. dead bug alternating leg lifts<br>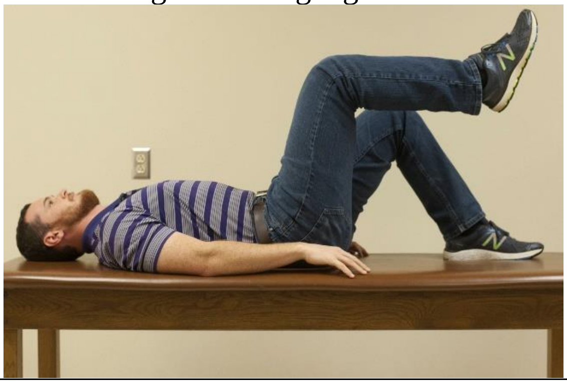
2<br><br>• day 1<br>• day 2<br>• day 3	1. transverse abdominis contractions<br>2. dead bugs with one hip held by hand in 90 degrees of flexion, alternating leg lifts<br>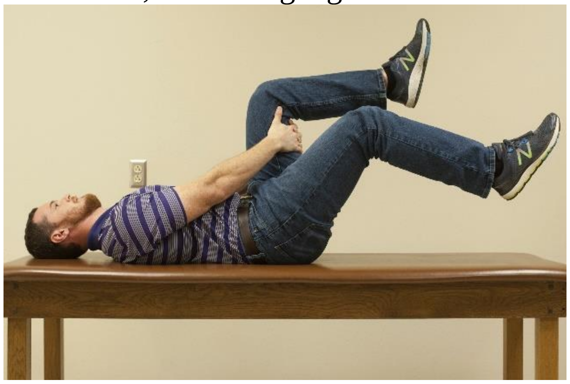<br>3. quadruped hip extensions

	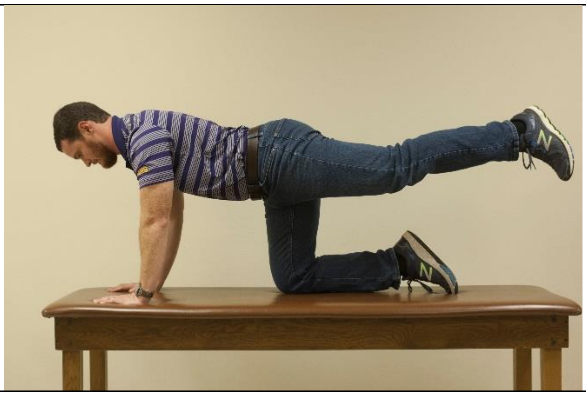
3<br><br>• day 1<br>• day 2<br>• day 2	1. transverse abdominis contractions seated on bosu ball<br>2. seated alternating marching (note: on exercises where the picture has the person sitting on a ball, you will be sitting on a chair, couch, bed, etc.).<br>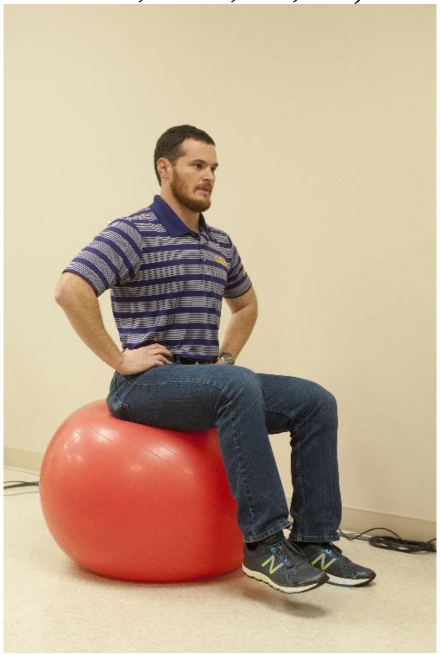<br>3. dead bugs with one hip held by hand in 90 degrees of flexion, alternating leg lifts.<br>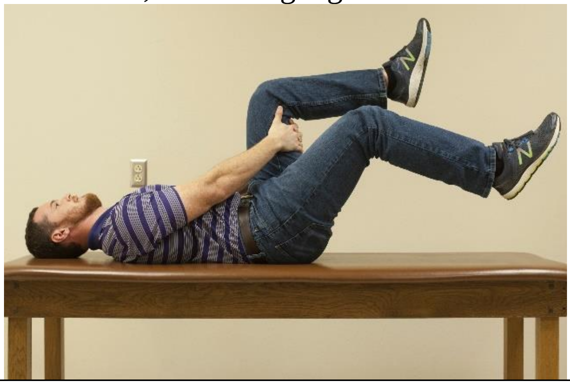

4<br><br>• day 1<br>• day 2<br>• day 3	1. seated alternating marching<br>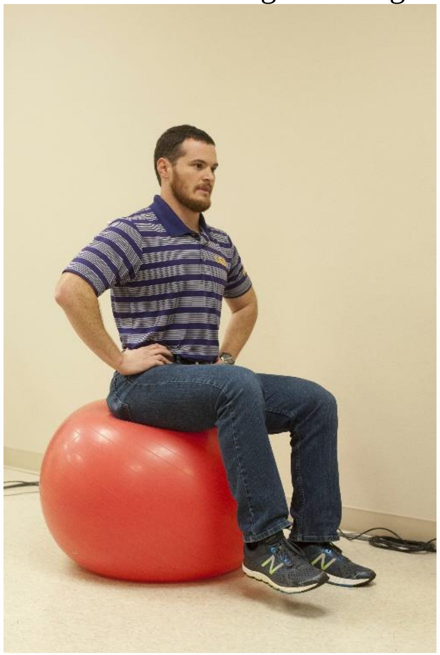<br>2. seated alternating knee extensions<br>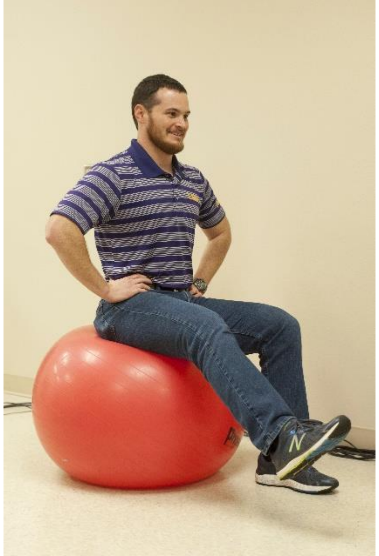<br><br>3. dead bugs with one hip actively maintained in 90 degrees of flexion, alternating leg lifts

	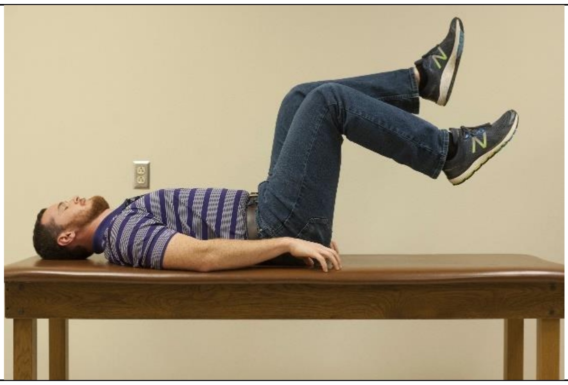
5<br><br>• day 1<br>• day 2<br>• day 3	1. seated alternating shoulder flexion and march on opposite arm/leg<br>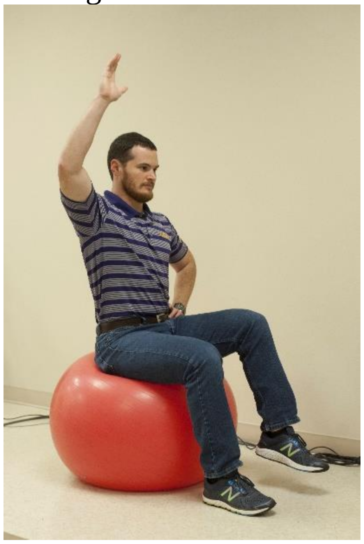<br>2. seated bilateral symmetrical d2 flexion/extension<br>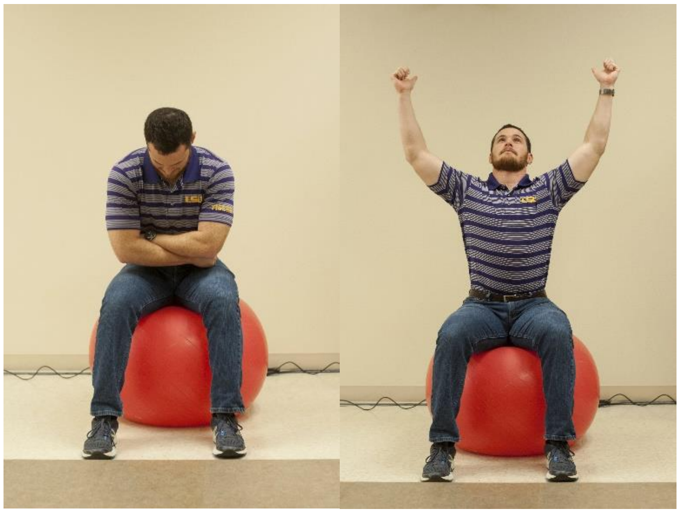<br>3. dead bug with both hips moving simultaneously through movements

	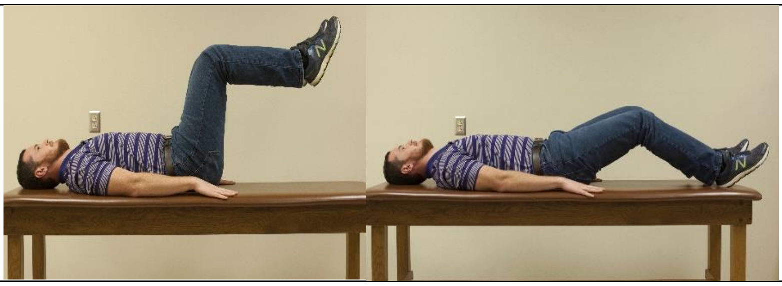
6<br><br>• day 1<br>• day 2<br>• day 3	1. seated bilateral asymmetrical d1/d2 flexion/extension<br>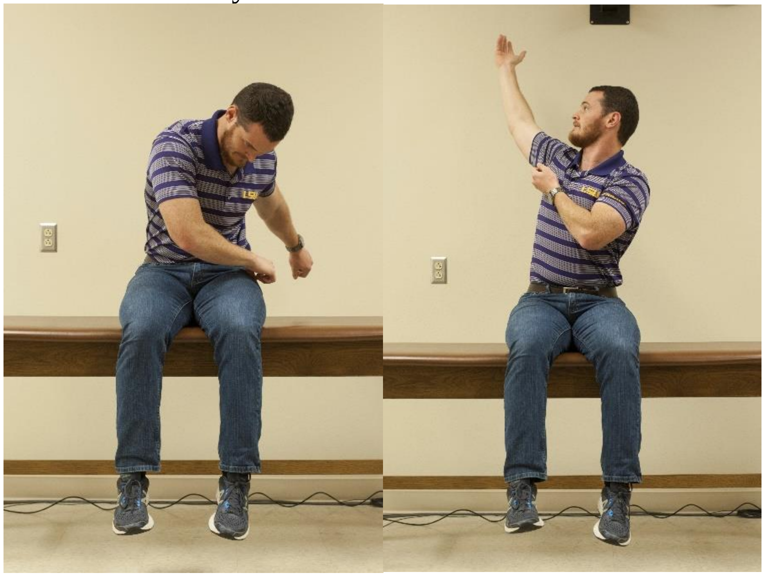<br>2. dead bug with both hips moving simultaneously through movements<br>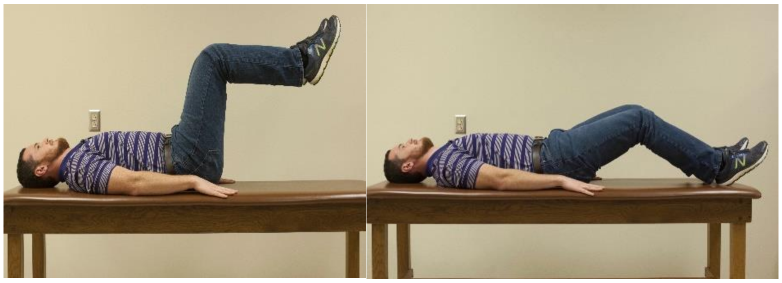<br>3. prone supermans

	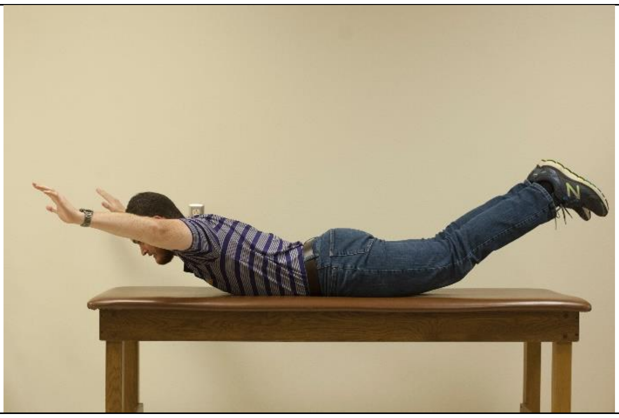

references:

kisner c, thorp jn. the spine: exercise and manipulation interventions. in: kisner c, colby la, eds. *therapeutic exercise: foundations and techniques.* 6th ed. Philadelphia, PA: F.A. Davis Company; 2012.
